# Effect of Low Level Laser Therapy on Chronic Compression of the Dorsal Root Ganglion

**DOI:** 10.1371/journal.pone.0089894

**Published:** 2014-03-04

**Authors:** Yi-Jen Chen, Yan-Hsiung Wang, Chau-Zen Wang, Mei-Ling Ho, Po-Lin Kuo, Mao-Hsiung Huang, Chia-Hsin Chen

**Affiliations:** 1 Department of Physical Medicine and Rehabilitation, Kaohsiung Medical University Hospital, Kaohsiung, Taiwan; 2 Institute of Clinical Medicine, College of Medicine, Kaohsiung Medical University, Kaohsiung, Taiwan; 3 Orthopaedic Research Center, College of Medicine, Kaohsiung Medical University, Kaohsiung, Taiwan; 4 School of Dentistry, College of Dental Medicine, Kaohsiung Medical University, Kaohsiung, Taiwan; 5 Department of Physiology, College of Medicine, Kaohsiung Medical University, Kaohsiung, Taiwan; 6 Graduate Institute of Medicine, School of Medicine, Kaohsiung Medical University, Kaohsiung, Taiwan; 7 Department of Medical Research, Kaohsiung Medical University Hospital, Kaohsiung Medical University, Kaohsiung, Taiwan; 8 Department of Physical Medicine and Rehabilitation, Faculty of Medicine, School of Medicine, Kaohsiung Medical University, Kaohsiung, Taiwan; 9 Department of Physical Medicine and Rehabilitation, Kaohsiung Municipal Ta-Tung Hospital, Kaohsiung, Taiwan; MGH, MMS, United States of America

## Abstract

Dorsal root ganglia (DRG) are vulnerable to physical injury of the intervertebral foramen, and chronic compression of the DRG (CCD) an result in nerve root damage with persistent morbidity. The purpose of this study was to evaluate the effects of low level laser therapy (LLLT) on the DRG in a CCD model and to determine the mechanisms underlying these effects. CCD rats had L-shaped stainless-steel rods inserted into the fourth and fifth lumbar intervertebral foramen, and the rats were then subjected to 0 or 8 J/cm^2^ LLLT for 8 consecutive days following CCD surgery. Pain and heat stimuli were applied to test for hyperalgesia following CCD. The levels of TNF-α, IL-1β and growth-associated protein-43 (GAP-43) messenger RNA (mRNA) expression were measured via real-time PCR, and protein expression levels were analyzed through immunohistochemical analyses. Our data indicate that LLLT significantly decreased the tolerable sensitivity to pain and heat stimuli in the CCD groups. The expression levels of the pro-inflammatory cytokines TNF-α and IL-1β were increased following CCD, and we found that these increases could be reduced by the application of LLLT. Furthermore, the expression of GAP-43 was enhanced by LLLT. In conclusion, LLLT was able to enhance neural regeneration in rats following CCD and improve rat ambulatory behavior. The therapeutic effects of LLLT on the DRG during CCD may be exerted through suppression of the inflammatory response and induction of neuronal repair genes. These results suggest potential clinical applications for LLLT in the treatment of compression-induced neuronal disorders.

## Introduction

Lower back pain with sciatica is a common symptom associated with many diseases of the lumbosacral spine, such as herniated intervertebral disc and degenerative disc diseases, and can result in functional disability. One of the major causes of this symptom is a narrowing of the intervertebral foramen accompanied by compression of the dorsal root ganglion (DRG) [1]. The DRG is unique in that it has bi-directional afferent branches that extend both to the periphery and into the spinal cord. The DRG is vulnerable to a variety of injuries, including direct compression and traction [2]. One of the major causes of pain in degenerative lumbar spinal disease is mechanical compression of the DRG, which can lead to molecular-based irritation involving the localized release of inflammatory cytokines [3]. Two of the primary cytokines responsible for the hyperalgesia observed in lumbar spinal diseases are tumor necrosis factor alpha (TNF-α) and interleukin-1 beta (IL-1β) [4]. It has been demonstrated that herniated disc tissues release IL-1β, which affects the somatosensory neural response at the dorsal root level [5]. Previous studies have also shown that TNF-α in the nucleus pulposus plays an important role in radicular pain and that sensory neurons display increased sensitivity to TNF-α in a rat CCD model [6], [7].

Growth-associated protein-43 (GAP-43) is involved in neuronal regeneration, and GAP-43 levels can be used as an indicator of nerve regeneration because its expression is correlated with the frequency of nerve sprouts [8]. Furthermore, this protein is exclusively localized to nerve fibers in the developing and regenerating adult peripheral nervous system [9]. Thus, GAP-43 immunoreactivity is often used to determine the efficacy of treatments aimed at inducing neuronal regeneration.

The chronic compression of the DRG (CCD) model established by Hu and Xing [10] is widely used in studies of neural responses and pain-related behaviors [11]. A previous study by Watanabe et al. also found that the CCD model led to allodynia as well as pain-related behaviors and structural changes within the spinal cord [12]. Localized treatment of mechanically compressed DRG – for example, using corticosteroids, lidocaine or a TNF-α antagonist – has been shown to reduce mechanical allodynia and thermal hyperalgesia and attenuate pain-related behaviors [13]–[15]. However, localized spinal injections are considered an invasive procedure. On the other hand, low level laser therapy (LLLT) exerts its biological effects through non-thermal mechanisms [16]. Previous studies have shown positive biological effects of LLLT in the peripheral nervous system, including the acceleration of regenerative processes following nerve injury as well as functional improvements [17], [18]. However, there have been relatively few publications on this topic, and no comprehensive studies have been carried out to characterize the mechanisms underlying these effects of LLLT for relieve neuropathic pain in a CCD model.

In the present study, we aimed to determine the effects of LLLT treatment of the DRG in a CCD model and evaluate its effectiveness in relieving neuropathic pain and changing behavioral patterns. The mechanisms underlying the effects of LLLT treatment were also investigated.

## Materials and Methods

### Animal preparation

A total of 36 male Sprague–Dawley rats (aged 8 weeks; 200–350 g) were used in this study. All animal experiments conformed to the regulations of the Animal Research Center of Kaohsiung Medical University and were approved by the Institutional Animal Care and Use Committee (IACUC. KMU no. 98020). The rats were randomly divided into 2 groups: a sham control group without CCD (Control group, n = 12) and the experimental groups with CCD, which was further randomly divided into 2 sub-groups, which were treated with LLLT at 0 J/cm^2^ (CCD group, n = 12) or 8 J/cm^2^ (CCD+LLLT group, n = 12) for 4 or 8 consecutive days following CCD. Half of the rats from each group were sacrificed 4 days post-CCD surgery. The other half of the rats from each group were sacrificed 8 days post-CCD surgery.

### Surgical procedure: dorsal root ganglion compression model

**Figure 1:**
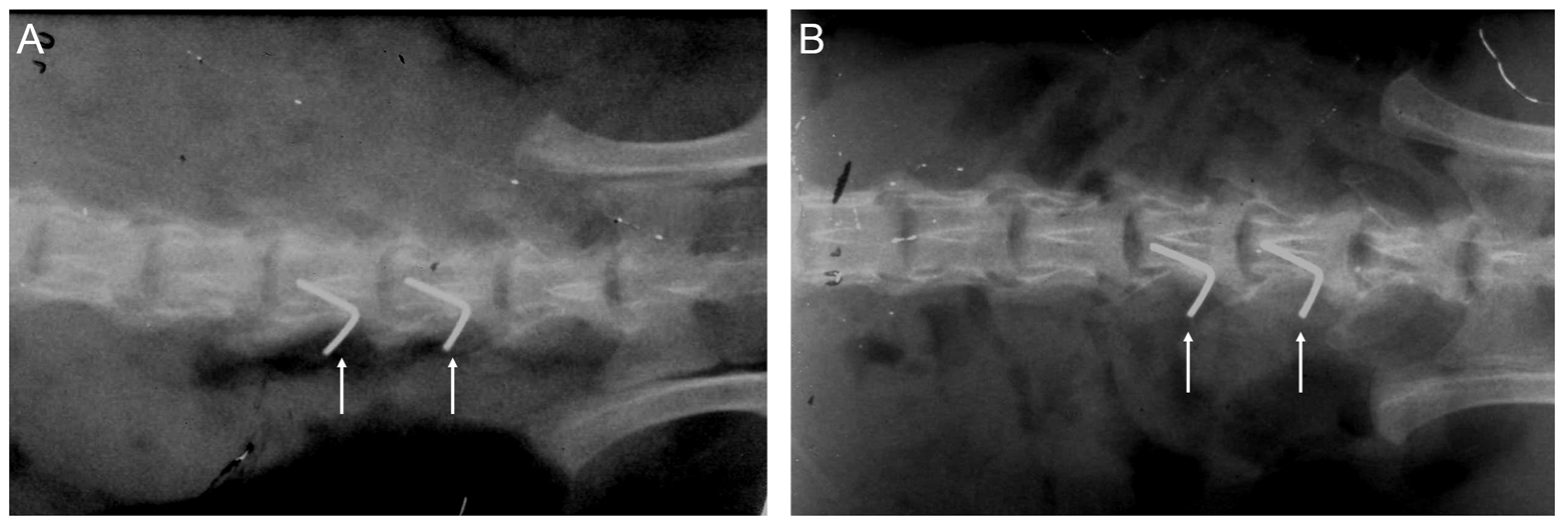
A soft X-ray radiographic analysis of CCD, both immediately following CCD surgery (A) and 8 days post-surgery (B); the arrows indicate the position of the L-shaped needles.

The animal model for DRG compression was generated as previously described [10]. The rats were anesthetized with ketamine (50 mg/kg) and xylazine (10 mg/kg) via the intraperitoneal route, and sterile surgical procedures were carried out. The skin was incised at the left lumbar vertebrae between L4 and L6. The left paraspinal muscles were separated from the mammillary process and the transverse process at the L4–L6 level. To exert chronic compression on the DRG, the L4 and L5 intervertebral foramen was cleanly exposed, and a fine, L-shaped needle (∼0.6 mm in diameter) was inserted ∼4 mm into the foramen. The needle was inserted at a 30° angle with respect to the dorsal middle line and a 10° angle with respect to the vertebral horizontal line. To determine when the needle tip reached the DRG, the hind leg muscles on the operated side of the animals were observed to detect a slight, transient twitch. Next, the needle was withdrawn from the foramen, and a stainless steel rod (4 mm in length and 0.5–0.8 mm in diameter) was inserted along the path of the needle. Finally, the muscular layer and skin were sutured, and antibiotics were administered following the surgical procedure. This treatment was meant to produce a steady compression against the L4 and L5 DRG (Fig. 1). Following the CCD surgical procedure and prior to sample harvesting, the position of the L-shaped needle was confirmed by obtaining soft X-rays (SOFTEX, Model M-100, Japan) at 43 KVP and 2 mA for 3.5 s.

### Low level laser therapy (LLLT)

The light source used in this study to induce low level laser irradiation was a Ga-Al-As laser with a wavelength of 808±5 nm and a power of ≦300 mW (TRANSVERSE IND. CO., LTD., Taipei, Taiwan). The laser dose selection was based on our previous study on the sciatic nerve crush injury (unpublished data) and review of other related articles [19]. The laser irradiation parameters are listed in Table 1. One day after CCD surgical procedure, the rats were treated with low level laser irradiation at the surgical site at the level of the L4 and L5 DRG. A targeted fluence of 8 J/cm^2^, measured by a laser power meter, was applied on rat dorsal ganglia daily for the LLLT group. The rats were sacrificed after 8 consecutive days of low level laser treatment, and the L4 and L5 DRG were harvested. For immunofluorescence, the L4 and L5 DRG were embedded in optimal cutting temperature (OCT) compound, after which the samples were stored at −80°C until sectioning. For RNA isolation, the L4 and L5 DRG were stored at −80°C until RNA isolation.

**Table 1:** The Laser Irradiation Parameters.

Laser type	Gallium Aluminum Arsenide (Ga-Al-As) semiconductor diode laser
Laser mode	Continuous wave
Mean power output (mW)	190
Beam area (cm^2^)	≦0.5
Power density (mW/cm^2^)	380
Fluence at the targeted area (J/cm^2^)	8
Transcutaneous fluence (J/cm^2^)	72
Anatomical location	L4 and L5 dorsal roots
Mode of application	Transcutaneous irradiation*
Irradiated area (cm^2^)	0.5
Irradiation time per point (sec)	207
Irradiation treatment	once a day

* The irradiation time was calculated according to the penetration rate measured by power meter. The penetration rate to DRG area is 11%.

### Behavioral pattern testing

The rats were stimulated with mechanical and thermal stimuli to determine their allodynia and hyperalgesia thresholds after 4 or 8 consecutive days of low level laser treatment. Mechanical allodynia (Ugo Basile, Italy) was assessed by measuring the foot withdrawal threshold associated with mechanical stimulation of the hind paw on the surgical side. A rat was placed in a transparent box and allowed to calm down. A metallic probe weighing 50 g was used to provide the mechanical stimulus. The intensity of the mechanical stimulus applied to the surgical-side hind paw was gradually increased until the paw was withdrawal by the animal, and the weight of the applied stimulus was recorded. The quantitative method employed for assessing mechanical allodynia was modified from the method described by Chaplan et al. [20] Heat hyperalgesia (Ugo Basile, Italy) was assessed using a modified version of the method described by Hargreaves et al [21]. After a rat was habituated to the transparent box, a mobile radiant heat source was placed under the table and focused on the surgical-side hind paw. Paw withdrawal latencies were recorded in seconds, and the heat source was set to stop heating after 20 s to avoid heat-induced injury. The heat stimulation was repeated 4 times, with an interval of more than 2 minutes, and the mean latency was calculated.

### RNA preparation and RT-PCR

Total RNA was isolated from the DRG using the TRIzol reagent (Gibco BRL, Bethesda, MD), then separated with chloroform (J.T. Backer) and precipitated with isopropanol (J.T. Backer). The obtained RNA pellet was washed once with 75% ethanol and once with 100% ethanol to ensure complete dehydration. The RNA was then dissolved in diethylpyrocarbonate-treated water, and its concentration was determined by measuring the absorbance at 260 and 280 nm using a spectrophotometer (ND-1000, NanoDrop). First-strand cDNA was reverse-transcribed using 1 µg of RNA template, Moloney murine leukemia virus reverse transcriptase and appropriate oligo-dT primers. Quantitative real-time polymerase chain reaction (PCR) was performed using a Bio-Rad iQ5 real-time detection machine (Bio-Rad Laboratories Inc., Hercules, CA). The reactions were carried out in a final volume of 12.5 µl containing the cDNA template, forward and reverse primers for each gene and SYBR® Green Real-time PCR Master Mix (TOYOBO) follow our previous study [22]. The primers used for amplification were listed in Table 2. Relative mRNA expression levels were calculated based on the threshold cycle (Ct) value for each PCR product and normalized to the levels of the housekeeping gene GAPDH through the comparative Ct method.

**Table 2:** Primer sequences used for Real-Time PCR.

Gene	Primer sequence
IL-1β	Forward: 5′-TCTCACAGCAGCATCTCGAC-3′
	Reverse: 5′-GGTCGTCATCATCCCACGAG-3′
TNF-α	Forward: 5′-GACCCCTTTATCGTCTACTCC-3′
	Reverse: 5′- GCAATCCAGGCCACTACTTC -3′
GAP43	Forward: 5′-GCCAAGGAGGAGCCTAAACA-3′
	Reverse: 5′-CTGCTTTCTGCAGTCTCCGT-3′
GAPDH	Forward: 5′-GAGAGAGGCCCTCAGTTGCCTGAGA-3′
	Reverse: 5′-GGCCCCTCCTGTTGTTATGGGG-3′

IL-1β: interleukin-1β; TNF-α: tumor necrosis factor alpha; GAP43: nerve growth-associated protein 43; GAPDH: Glyceraldehyde 3-phosphate dehydrogenase.

### Immunohistochemistry (IHC) and immunofluorescence

Frozen DRG sections (8 µm) were fixed with 4% paraformaldehyde for 30 min. Following blocking with 5% bovine serum albumin, the DRG sections were incubated with anti-TNF-α (Millipore; 1∶100) and anti-GAP43 (Millipore; 1∶100) antibodies overnight at 4°C. Following 3 washes in 1× PBS, the sections were incubated with an anti-rabbit Alexa Fluor 594-conjugated secondary antibody (Molecular Probe; for TNF-α, 1∶200) or an anti-mouse FITC-conjugated secondary antibody (Dako, for GAP-43, 1∶200) for 1 hour at room temperature. The sections were then washed twice in 1× PBS and incubated for 5 min with 4′,6-diamidino-2-phenylindole (DAPI; 10 µg/ml; Sigma) for nuclear counterstaining, followed by 3 additional washes with 1× PBS. Finally, the sections were dried, mounted and observed through fluorescence microscopy (Nikon, Eclipse, TE300). After staining, images of samples from three independent experiments were captured on a fluorescence microscope. Ratios of fluorescent signal were measured from three random of microscopic areas (18548.61 µm^2^ per field) [23]. Images were captured using a digital camera (EvolutionTM VF) and analyzed with Image-Pro Plus® software (Media Cybernetics, Silver Spring, MD).

### Statistical analysis

SPSS version 17.0 was used for statistical analyses. The results are presented as mean ± standard deviation values. Statistically significant differences were determined by applying Student's t test and analysis of variance (ANOVA), followed by the post-hoc Tukey's test for multiple comparisons; p-values less than 0.05 were considered to be statistically significant.

## Results

### LLLT reduces CCD-mediated mechanical and thermal hyperalgesia

**Figure 2:**
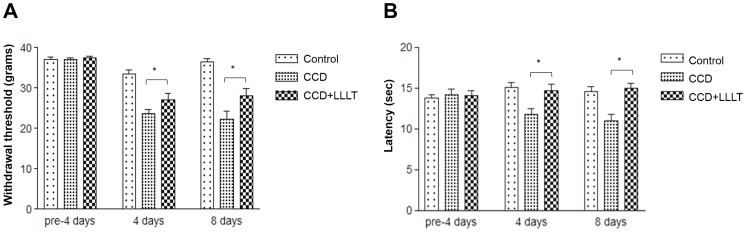
LLLT reduced CCD-mediated mechanical and thermal hyperalgesia. The behavioral patterns of rats in the control, CCD and CCD+LLLT groups were monitored at the indicated times (n = 6). (A) Foot withdrawal threshold responses to mechanical stimuli. (B) Foot withdrawal latencies in response to thermal stimuli. The levels of statistical significance are as follows: *, p<0.05.

To verify the pain relief effects of LLLT, rats with CCD were treated with or without LLLT and monitored for the behavioral patterns associated with mechanical and thermal hyperalgesia. In the CCD group not subjected to laser irradiation, both the mechanical and thermal thresholds were significantly decreased after 4 and 8 days of compression compared to the Control group (Fig. 2A and 2B). In comparison to the CCD group, the CCD+LLLT group which was subjected to 8 J/cm^2^ of laser irradiation showed significant increases in both the mechanical and thermal thresholds after 4 and 8 days of laser treatment, although the increase in the mechanical threshold was not as great as in the Control group (Fig. 2A and 2B). These findings indicate that LLLT effectively improved pain tolerance following chronic DRG compression.

### LLLT suppresses the CCD-induced expression of inflammatory cytokines

**Figure 3:**
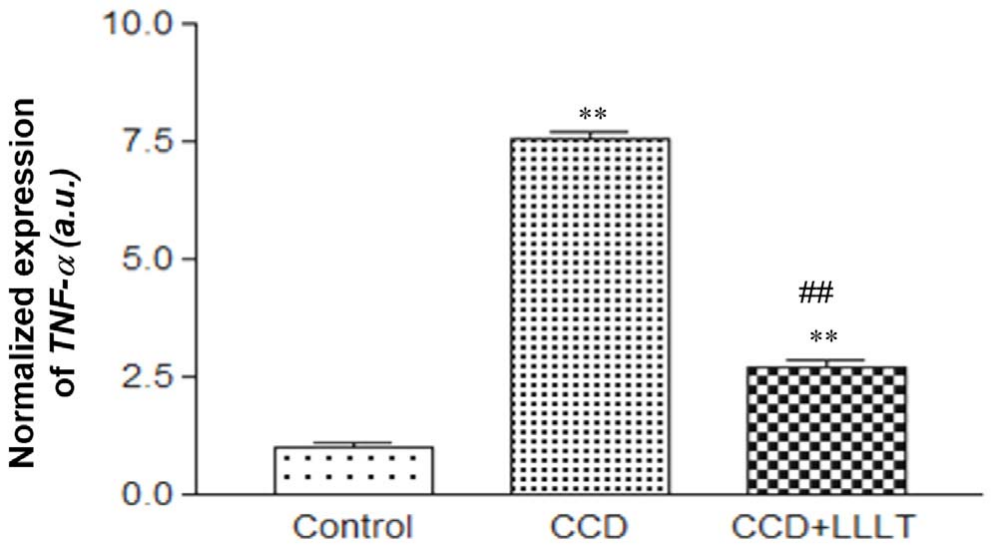
LLLT suppressed the mRNA expression level of CCD-induced *TNF*-α. The level of *TNF*-α mRNA expression was analyzed using the 2^−ΔCT^ method and normalized to the Control group. The levels of statistical significance are as follows: **, p<0.01 relative to the Control group; ##, p<0.01 relative to the CCD group.

The inflammatory response is important for CCD-mediated hyperalgesia, and previous studies by our group and others have shown that LLLT may reduce this response [22], [24]. Therefore, we investigated whether LLLT decreases the expression of inflammatory cytokines through real-time PCR analysis. Following DRG compression for 8 days, the levels of TNF-α and IL-1β mRNA of the CCD group were significantly increased within the DRG compared with the Control group (p<0.05). Interestingly, in the CCD+LLLT group, the mRNA levels of both TNF-α (Fig. 3) and IL-1β (Fig. S1) were significantly decreased within the DRG compared to the CCD group (p<0.05).

**Figure 4:**
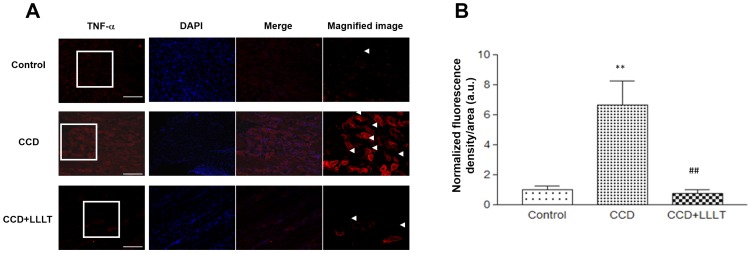
LLLT suppressed the protein expression levels of CCD-induced inflammatory cytokines. (A) Fluorescence images of TNF-α staining from the Control and CCD groups with or without LLLT were analyzed. Representative images from the Control (*upper row*), CCD (*middle row*) and CCD+LLLT (*bottom row*) groups are shown. TNF-α signals are shown in the *left column* (red). Images of nuclear staining with DAPI are presented in the *middle-left column* (blue), and merged fluorescence images are shown in the *middle-right column*. Magnified image of TNF-α are provided in the *right column* (magnification 400×) and representative signals are indicated by *arrowheads*. *Scale bars*, 10 µm. (B) A graph displaying the quantification of TNF-α fluorescence. The levels of statistical significance are as follows: **, p<0.01 relative to the Control group; ##, p<0.01 relative to the CCD group.

We analyzed the expression of the TNF-α protein via immunofluorescence staining, as shown in Figure 4A, and we quantified the density of anti-TNF-α staining within the DRG (Fig. 4b). In the CCD group, TNF-α protein expression was significantly higher than in the Control group (p<0.01). Following LLLT, TNF-α protein expression in the CCD+LLLT group was significantly reduced compared with the CCD group (p<0.01) (Fig. 4B). These findings indicate that chronic DRG compression induced an inflammatory reaction and that LLLT had a beneficial effect regarding reducing the inflammatory response following chronic DRG compression.

### LLLT enhances GAP-43 expression in the CCD model

**Figure 5:**
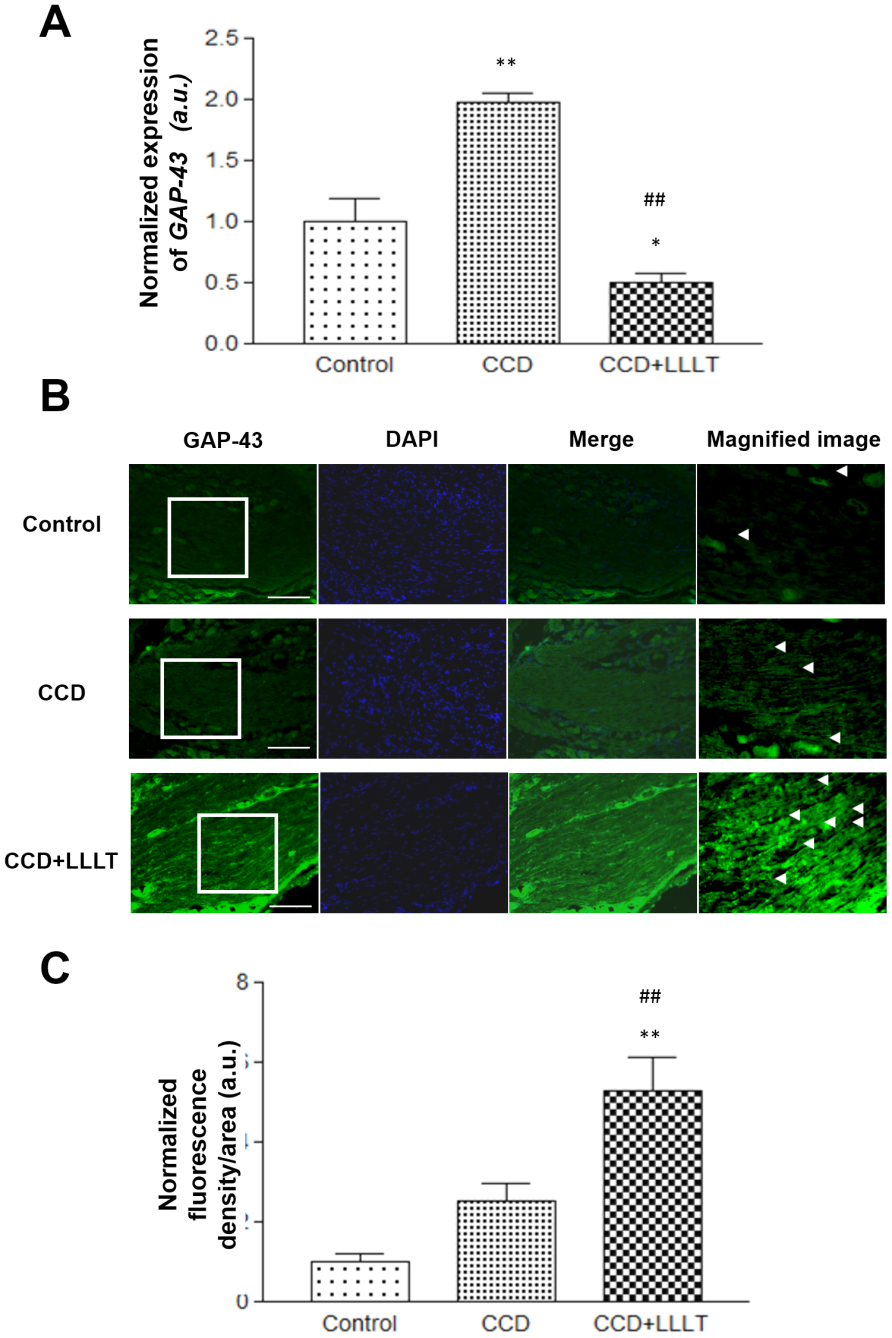
LLLT increased *GAP-43* expression in a CCD model. (A) The levels of *GAP-43* mRNA expression were analyzed using the 2^−ΔCT^ method and normalized to the Control group. (B) Fluorescence images of GAP-43 staining from the Control or CCD groups with or without LLLT were analyzed. Representative images from the Control (*upper row*), CCD (*middle row*) and CCD+LLLT (*bottom row*) groups are shown. *GAP-43* signals are presented in the *left column* (green). Images of nuclear staining with DAPI are provided in the *middle-left column* (blue), and merged fluorescence images are shown in the *middle-right column* Magnified image of *GAP-43* are provided in the *right column* (magnification 400×) and representative signals are indicated by *arrowheads*. *Scale bars*, 10 µm. (C) A graph displaying the quantification of *GAP-43* fluorescence. The levels of statistical significance are as follows: *, p<0.05 and **, p<0.01 relative to the control group; #, p<0.05 and ##, p<0.01 relative to the CCD groups.

To investigate whether LLLT enhances nerve repair in chronically compressed DRG, the expression of GAP43 was monitored via real-time PCR analysis. Following DRG compression for 8 days in the CCD group, GAP-43 mRNA levels were increased within the DRG compared to the Control group (p<0.01). Following LLLT, GAP-43 mRNA levels were significantly decreased within the DRG of the CCD+LLLT group compared to the CCD group (p<0.01) (Fig. 5A). We also analyzed the expression of the GAP43 protein via immunofluorescence staining (Fig. 5B) and quantified the density of anti-GAP43 staining within the DRG (Fig. 5C). In the CCD group, the density of anti-GAP43 staining within the DRG was higher than in the Control group, although this difference was not significant. However, following LLLT, the density of anti-GAP43 staining was significantly increased compared with the sham Control and CCD without laser irradiation groups (p<0.01) (Fig. 5C).

## Discussion

The present study demonstrated that non-invasive LLLT (8 J/cm^2^) significantly reduced CCD-mediated mechanical and thermal hyperalgesia in a rat model. LLLT targeting the DRG in a CCD model not only reduced pro-inflammatory cytokine expression, but also promoted neural regeneration.

Radicular pain following disc herniation is thought to be caused by mechanical nerve root compression as well as by chemical factors involved in inflammation that are induced by the direct contact of the nucleus pulposus with nerve fibers. Mechanical compression of herniated intervertebral discs induces the inflammatory process and the release of specific cytokines, and it plays a fundamental role in the development of hyperalgesia [4], [25]. IL-1β is a potent pro-inflammatory cytokine that stimulates the release of other inflammatory mediators and causes hyperalgesia [5], and TNF-α is involved in the initiation of neuropathic pain through activation of p38 MAPK [26]. Furthermore, it has been demonstrated that cytokine inhibition decreases hyperalgesia in animal models [4], [15]. In the present study, direct DRG compression was found to result in increased levels of IL-1β and TNF-α, and behavioral tests revealed mechanical and thermal hyperalgesia. These findings suggest that increased levels inflammatory cytokines contribute to hyperalgesia in animal models of chronic dorsal root compression.

LLLT has been widely applied in clinical practice to facilitate nerve regeneration and relieve pain. In vivo studies addressing peripheral nerve injury, particularly those involving sciatic nerve injuries in rats, have shown that the application of low power laser irradiation to injured nerves induces histological and morphological changes and improves functional recovery [27]–[29]. Similarly, Hsieh et al. demonstrated that LLLT significantly increased the rat paw withdrawal threshold following chronic constriction sciatic nerve injury and that the expression of TNF-α and IL-1β was significantly reduced following LLLT [24].

An in vitro study involving laser stimulation of cultured murine DRG cells demonstrated that low level laser irradiation suppresses action potentials induced by Bradykinin, suggesting that the LLLT may block the conduction of nociceptive signals in primary afferent neurons [30]. However, the application of LLLT to compressed dorsal root ganglia has not been well investigated, and studies addressing the effects of LLLT on the DRG for the purpose of relieving neuropathic pain are not common in the literature. In the present study, a rat CCD model was used to evaluate the effects of LLLT applied directly to the injured DRG. The levels of the inflammatory cytokines TNF-α and IL-1β were significantly reduced after 8 days of LLLT. Furthermore, behavioral tests showed that hyperalgesia in response to both mechanical and thermal stimuli were reduced after only 4 consecutive days of LLLT. These findings suggest that LLLT applied to chronically compressed dorsal root ganglia may alleviate neuropathic pain by reducing the inflammatory response. However, our data did not show time dependency of functional recovery after LLLT. Our previous study showed that LLLT can suppress inflammation response by modulating intracellular cyclic AMP level and NF-kappaB activity in a very short time (≦24 h) [22]. We demonstrated that LLLT suppresses the CCD-induced expression of inflammatory cytokines in Fig. 3. One of the possible reasons is LLLT decreased inflammation response and swelling around compressed DRG within 4 days, which effectively decreased the mechanical and thermal thresholds in the CCD+LLLT group. Therefore, further work will be necessary to observe the functional recovery in day 1 to day 4 after CCD and to determine the correlation between functional recovery and the expression levels of inflammatory cytokines.

GAP-43 is a neuronal growth cone marker that localizes exclusively to nerve fibers, and it is present in regenerating peripheral nerves [27]. It was previously proposed by Shin et al. that LLLT may have an effect on nerve regeneration. These authors observed increased GAP-43 immunoreactivity in regenerating peripheral nerves following laser treatment with a 605 nm, 5 mW continuous laser beam for 5 consecutive days immediately following sciatic nerve injury in rats; the intensity of GAP-43 immunoreactivity first began to differ between the irradiated and non-irradiated groups 1 week after injury, peaked after 3 weeks and declined after 5 weeks in both groups [27]. In the present study, we applied low level laser irradiation to injured DRG for 8 consecutive days immediately following dorsal root compression in rats. Immunofluorescence assays were performed to observe the GAP-43 staining intensity following DRG injury. We observed a significant increase in GAP-43 protein expression by day 8 post-treatment in the CCD+LLLT group compared with the CCD alone group. Interestingly, the expression of GAP-43 mRNA in the CCD+LLLT groups was lower than in the CCD group. These results suggest that LLLT may induce GAP-43 expression in a rapid manner. Following GAP-43 induction and neuronal recovery, the levels of GAP-43 expression eventually declined, which may be related to the highly regulated nature of GAP-43 during neuro-regeneration.

Although more neuronal markers should be stained to further confirm what type of cells or where in a spinal cord neurons/glial cells express TNF-α and GAP-43. The cellular morphology analysis of immunofluorescence showed that TNF-α majorly expressed in neuron cells. GAP-43 is a kind of secreting protein which distributes in neurons and nerve fibers; we can find that GAP-43 is stained in cells and ECM. The expression levels of TNF-α was reduced by the application of LLLT. And, the expression of GAP-43 was enhanced by LLLT. These findings demonstrate a beneficial effect of LLLT during the early stages of neuronal regeneration, and they suggest that low power laser therapy should be applied immediately following nerve injury.

Positive biological effects of LLLT have been reported with respect to neural regeneration and functional recovery following peripheral nerve injury [14]. Furthermore, a possible mechanism by which low level laser treatment might alleviate pain has been proposed. Chow et al. studied the effects of an 830 nm laser on cultured rat DRG neurons, and they reported that irradiation induced reversible axonal varicosities, reduced the mitochondrial membrane potential and blocked fast axonal flow in small- and medium-diameter rat DRG neurons, thus providing several potential mechanisms for laser-induced pain relief [31]. In a clinical setting, diseases of the lumbosacral spine are quite common, and disc herniation, degenerative disc diseases and spinal stenosis may directly compress dorsal root ganglia, leading to neuronal damage and radicular pain. Therefore, the use of laser-based therapies may serve as an effective, non-invasive means for enhancing neuronal regeneration and alleviating pain in these types of diseases.

In conclusion, the early application of low level laser irradiation to chronically compressed rat dorsal root ganglia decreased the levels of inflammatory cytokines and facilitated neural regeneration, as demonstrated by molecular analyses of TNF-α, IL-1β and GAP-43 levels. Application of LLLT also attenuated pain-related behaviors induced by DRG compression. These findings suggest that LLLT may be used to modulate the mechanical compression and molecular-based inflammation observed in lumbar disc diseases. In clinical practice, low level laser therapies may provide effective, conservative treatment options for the relief of sciatica caused by lumbar disc diseases.

## Supporting Information

Figure S1 LLLT suppressed the mRNA expression level of CCD-induced *IL-1β*. The level of *IL-1ß* mRNA expression was analyzed using the 2^−ΔCT^ method and normalized to the Control group. The levels of statistical significance are as follows: *, p<0.05 and **, p<0.01 relative to the Control group; #, p<0.05 and ##, p<0.01 relative to the CCD group.
(DOC)
