# Supplementary material for: Effect of Low Level Laser Therapy on Chronic Compression of the Dorsal Root Ganglion
Source: PLoS One. 2014 Mar 4;9(3):e89894. doi: 10.1371/journal.pone.0089894 (PMC3942382; doi:10.1371/journal.pone.0089894)

**Fig. S1** LLLT suppressed the mRNA expression level of CCD-induced *IL-1β*. The level of *IL-1β* mRNA expression was analyzed using the 2^-ΔCT^ method and normalized to the Control group. The levels of statistical significance are as follows: *, p<0.05 and **, p<0.01 relative to the Control group; #, p<0.05 and ##, p<0.01 relative to the CCD group.


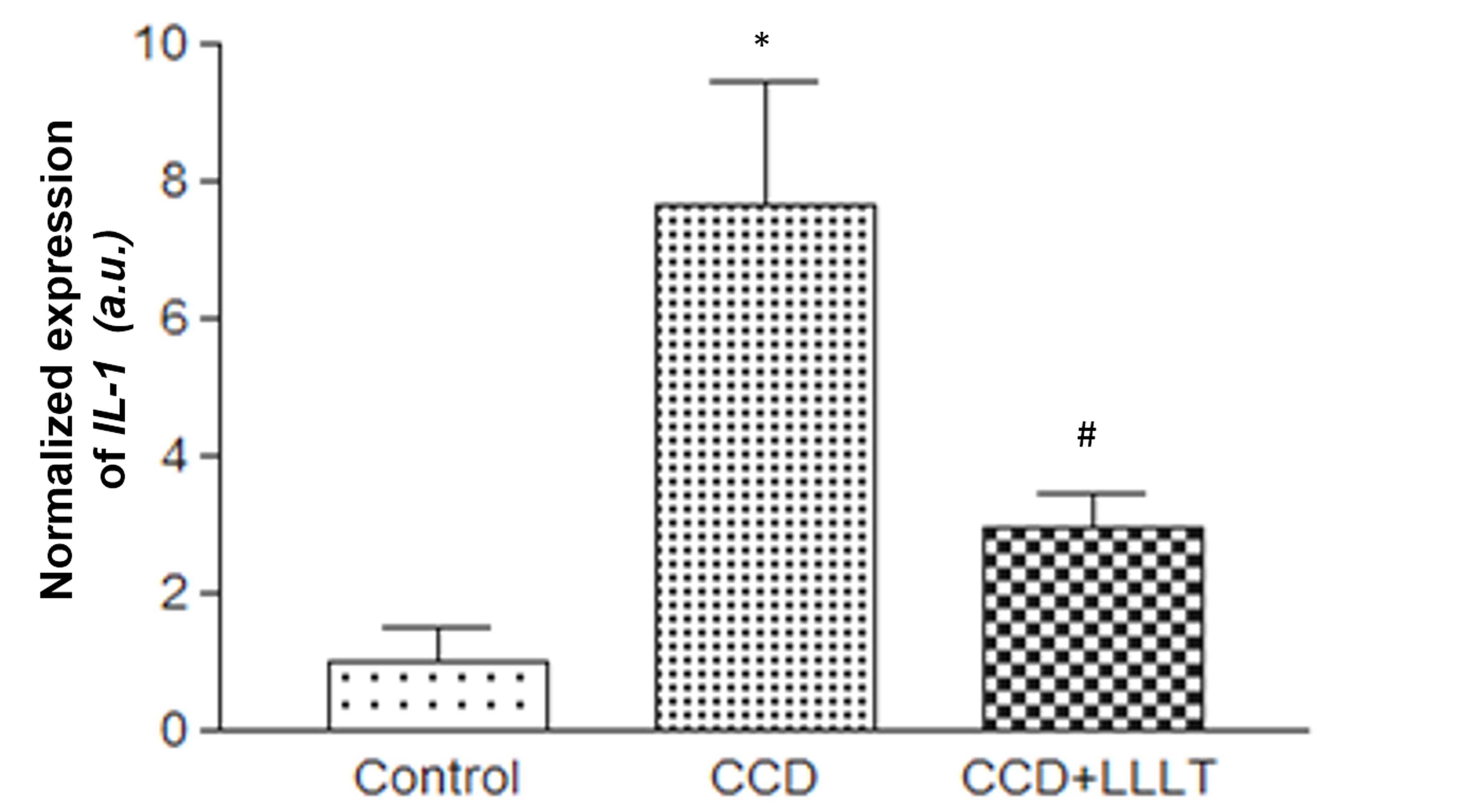

Supplement: Figure S1 — LLLT suppressed the mRNA expression level of CCD-induced IL-1β. The level of IL-1ß mRNA expression was analyzed using the 2−ΔCT method and normalized to the Control group. The levels of statistical significance are as follows: *, p<0.05 and **, p<0.01 relative to the Control group; #, p<0.05 and ##, p<0.01 relative to the CCD group. (DOC) [file pone.0089894.s001.doc]
